# Outcomes of Use of Inotropes at Waitlisting Through Heart Transplantation: The UNOS Experience

**DOI:** 10.3390/jcdd12090364

**Published:** 2025-09-17

**Authors:** Marco Gemelli, Ilias P. Doulamis, Thanakorn Rojanathagoon, Aspasia Tzani, Athanasios Rempakos, Polydoros Kampaktsis, Alvise Guariento, Ernesto Ruiz Dunque, Rabea Asleh, Paulino Alvarez, Vincenzo Tarzia, Gino Gerosa, Alexandros Briasoulis

**Affiliations:** 1 Bristol Heart Institute, University of Bristol, Bristol BS2 8ED, UK; fb25286@bristol.ac.uk; 2 Department of Surgery, Lahey Clinic, Burlington, MA 01805, USA; 3 School of Medicine, University of Nottingham, Nottingham NG7 2RD, UK; 4 Brigham and Women’s Hospital Heart and Vascular Center, Harvard Medical School, Boston, MA 02115, USA; 5 Medical School of Athens, National and Kapodistrian University of Athens, 10679 Athens, Greece; 6 Division of Cardiology, Columbia University Irving Medical Center, New York City, NY 10032, USA; 7 Department of Cardiac, Thoracic, Vascular Sciences and Public Health, University of Padua, 35128 Padua, Italy; 8 Division of Cardiovascular Medicine, Section of Heart Failure and Transplantation, University of Iowa, Iowa City, IA 52242, USA; 9 Department of Cardiovascular Diseases, Mayo Clinic, Rochester, MN 55905, USA; 10 Heart Institute, Hadassah University Medical Center, Hebrew University of Jerusalem, Jerusalem 9190500, Israel; 11 Division of Cardiology, Cleveland Clinic Foundation, Cleveland, OH 44195, USA

**Keywords:** UNOS, heart transplantation, MCS, inotropes, survival

## Abstract

Background: Despite its use in patients awaiting heart transplant (HT), the impact of continuous inotropic support on short-term complications and long-term transplant outcomes remains unclear. This study evaluated inotrope use at the time of HT on perioperative complications and post-transplant survival, comparing outcomes at 30 days, 1 year, and 10 years with mechanical circulatory support (MCS) strategies including ECMO, IABP, and VADs. Methods: A retrospective analysis of the United Network for Organ sharing (UNOS) registry was performed, stratifying patients based on bridge strategy at the time of transplant: inotropes, ECMO, IABP, or VADs. Baseline characteristics, perioperative complications, and 30-day, 1-year, and 10-year post-transplant survival outcomes were analyzed across groups. Survival was assessed using Kaplan–Meier and Cox proportional hazards models. Results: Among the 11,801 heart transplant patients included, 9330 were on inotropes, 372 were on ECMO, 1072 received an IABP, and 1027 had VADs. Inotrope-bridged patients had significantly lower 30-day and 1-year mortality rates compared to the ECMO, IABP, and VAD groups. They also experienced reduced incidences of post-transplant dialysis and stroke. At 10 years, the inotrope group demonstrated superior long-term survival, with significantly lower mortality risk compared to ECMO (HR: 1.81; CI: 1.49–2.20, *p* < 0.001), IABP (HR: 1.19; CI: 1.06–1.32, *p* = 0.005), and VAD (HR: 1.18; CI: 1.10–1.27, *p* < 0.001). Conclusions: Continuous use of inotropes after waitlisting is associated with lower short, intermediate, and long-term mortality and does not lead to worse outcomes compared to ECMO, IABP, and VAD support. When mechanical support is not an option, inotropic therapy remains a viable and effective strategy.

## 1. Introduction

Heart transplantation (HT) remains the ultimate treatment for end-stage heart failure (HF) [1], and recent changes in United States allocation policies, particularly the 2018 update aimed at improving access for high-urgency patients [2], have influenced clinical practices and indirectly increased reliance on short-term mechanical circulatory support (MCS) devices, raising concerns about clinical outcomes and policy exceptions [3,4,5,6]. For patients unable to receive MCS, inotropic therapy, using agents like dobutamine or milrinone to enhance cardiac function and stabilize hemodynamics, serves as a critical bridge to transplant [7]. However, the effects of continuous inotrope use on short-term complications and long-term outcomes are not well understood [8]. Therefore, our study aimed to assess the impact of inotropic support at the time of HT on perioperative complications and post-transplant survival, directly comparing outcomes at 30 days, 1 year, and 10 years with those of patients supported by MCS strategies, including ECMO, IABP, and VADs.

## 2. Materials and Methods

### 2.1. Ethical Statement and Study Design

This retrospective observational study was approved by the institutional ethics review board of the University of Iowa and adhered to the Strengthening and the Reporting of Observational Studies in Epidemiology reporting guidelines. The need for informed consent was waived because this was a secondary analysis of a de-identified dataset. The UNOS registry is a prospective registry which has collected data of patients undergoing organ transplant in the United States since 1987. Data includes information on every donor, candidates, and recipients of organ transplant, including baseline characteristics, operative data, and post-transplant follow-up. Patients transplanted before October 2018 were classified according to the “old” UNOS allocation system into 3 classes: 1A, 1B, and 2. Patients transplanted after 2018 were classified according to the “new” allocation system, which includes 6 different classes.

### 2.2. Patients and Outcomes

De-identified patient-level variables on all adult patients who underwent only heart transplantation between 2000 and 2021 were collected from the UNOS Registry. Baseline recipient characteristics included age, gender, race, body mass index (BMI), history of diabetes, cerebrovascular disease, smoking, malignancy, dialysis, abuse of intravenous (IV) drugs, prior cardiac surgery, and ICD implantation. Furthermore, our analysis included history of mechanical circulatory support (MCS) devices at listing and/or at transplantation, on ventilator at listing, creatinine level, and Calculated Panel Reactive Antibody (CPRA) value. Finally, we considered hemodynamic criteria and UNOS status according to the old or new allocation and number of days in each status level. Collected donor characteristics included age, race, gender, BMI, creatinine, history of diabetes, left ventricular ejection fraction (LVEF), ischemic time, and distance. Patients were divided into two groups according to their use or not of inotropes at the time of transplantation. Subsequently, multi-modality bridging analysis was performed, dividing the population into 4 groups: on inotropes, on ECMO, on IABP, or on LVAD. Patients receiving both inotropes and any mechanical circulatory support device were excluded from the sub-study. Follow-up data were available for mortality, rate of stroke, dialysis, permanent pacemaker implantation, and rejection requiring treatment.

### 2.3. Statistical Analysis

Normally distributed continuous variables were expressed as mean ± standard deviation, while categorical variables were expressed as frequencies and percentages. Unpaired Student’s *t*-test (inotropes vs. no inotropes) and one-way analysis of variance (ANOVA) (inotropes vs. ECMO, IABP, and LVAD) was performed for between-group comparisons of continuous variables, while a Pearson chi-squared test compared categorical variables. Also, post hoc pairwise comparisons (e.g., Tukey’s test) were performed to determine the difference between inotropes and other groups. For comparisons of categorical data involving more than two groups, post hoc pairwise chi-squared tests with Bonferroni correction was applied. Univariable logistic regression was used to compare binary outcomes among teams, while univariable Cox regression was performed to assess survival. All statistical analyses were performed with Stata (StataCorp. 2023. Stata Statistical Software: Release 18. College Station, TX, USA: StataCorp LLC.) A *p*-value of <0.05 was considered to indicate statistical significance of differences between the two groups.

## 3. Results

### 3.1. Patient Characteristics: Inotropes vs. Non-Inotropes

A total of 10,528 patients had data on inotropes use at the time of transplantation. A total of 68% of them (7168) were on inotropes, while the remaining 32% (3360) were not. Baseline characteristics were significantly different in the two groups: patients on inotropes at the time of transplantation were more frequently female (28% vs. 22%, *p* < 0.001), had higher rates of history of diabetes (27% vs. 25%, *p* = 0.024), malignancy (8% vs. 7%, *p* = 0.001), and prior cardiac surgery (24% vs. 23%, *p* = 0.005), and were more often on IABP at the time of transplantation (17% vs. 8%, *p* < 0.001). On the other hand, patients without inotropes at the time of surgery had a higher BMI (27 vs. 26, *p* < 0.001), higher rates of smoking history (38% vs. 34%, *p* < 0.001), dialysis (8% vs. 6%, *p* = 0.001), and IV drug addiction (16% vs. 12%, *p* < 0.001). Also, they were more frequently on IABP (15% vs. 12%, *p* = 0.001), on LVAD (7% vs. 2%, *p* < 0.001) and/or on ventilator (9% vs. 4%, *p* < 0.001) at listing and had higher PCWP (23 vs. 21 mmHg, *p* < 0.001) and mPAP (33 vs. 31 mmHg, *p* < 0.001).

**Figure 1:**
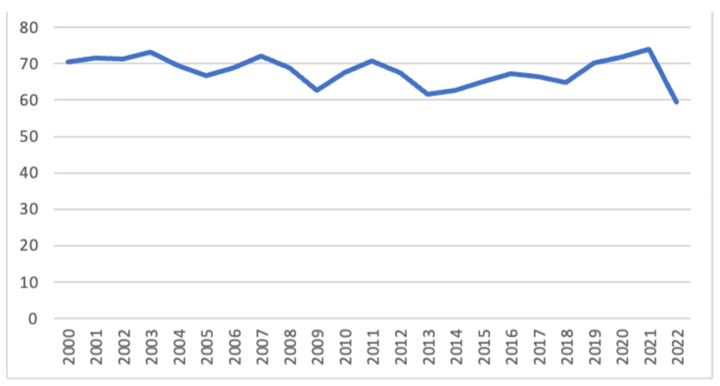
Trend of inotropes use (%) during the study period.

Donors of patients on inotropes at the time of transplantation were older (32 vs. 31 years, *p* = 0.031), of female gender (31% vs. 24%, *p* < 0.001), and had a history of diabetes (3% vs. 2%, *p* = 0.002). The distance between the harvesting center and the implantation center was significantly higher in patients on inotropes (202 vs. 178 miles, *p* < 0.001), but no significant differences in terms of ischemic time were found (3.3 vs. 3.2 h, *p* = 0.466) (Table 1). A decrease in inotrope use throughout the waitlist period was noted over time (b: −0.02, *p* < 0.001) (Figure 1).

### 3.2. Impact of Use of Inotropes on Survival

**Figure 2:**
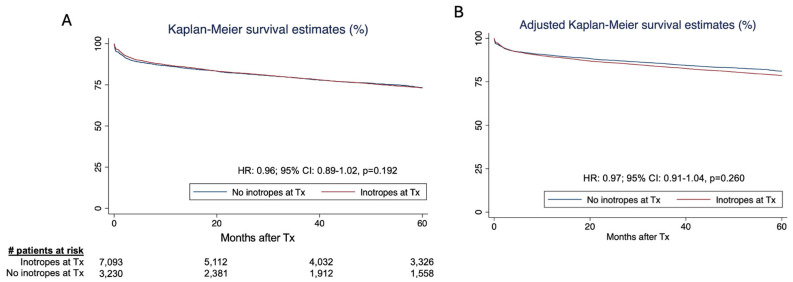
Kaplan–Meier curves representing unadjusted (**A**) and adjusted (**B**) long-term survival in those on inotropes vs. no inotropes at the time of heart transplant. CI: confidence interval. HR: hazard ratio.

No difference was noted in the inotropes vs. non-inotropes groups in terms of 1-year mortality (OR: 0.97; 95% CI: 0.86–1.09, *p* = 0.602) and survival at 5 years was similar (HR: 0.96; 95% CI: 0.89–1.02, *p* = 0.192) (Figure 2). After adjustment for gender, body mass index, smoking status, malignancy, dialysis, intravenous drug use, prior cardiac surgery, and mean pulmonary artery pressure, the hazard ratio for survival was 1.06 (95% CI: 1.02–1.09; *p* < 0.001). The unadjusted and adjusted Kaplan–Meier curves for 5-year survival are represented in Figure 2.

### 3.3. Impact of Use of Inotropes on Post-Operative Complications

Use of inotropes at the time of HT was associated with decreased 30-day mortality (OR 0.74; 95% CI: 0.62–0.88, *p* = 0.001) and the need for post-operative dialysis (OR: 0.88; 95% CI: 0.79–0.98, *p* = 0.017). No difference was found in terms of post-operative stroke (OR: 0.90; 95% CI: 0.72–1.12, *p* = 0.354) or post-operative permanent pacemaker implantation (OR: 0.85; 95% CI: 0.66–1.09, *p* = 0.20).

### 3.4. Patient Characteristics: Inotropes vs. ECMO, IABP, and VAD

In the multi-modality bridging analysis, a total of 11,801 adult patients undergoing HT were included in the analysis and stratified into four groups based in bridging modality at the time of transplant: inotropes 9330 (79.0%), ECMO 372 (3.2%), IABP 1072 (9.1%), and VADs 1027 (8.7%). Baseline characteristics differed significantly across groups: patients in the inotrope group were older on average (mean age of 51.3 years) compared to those supported with ECMO (46.3 years), IABP (50.7 years), or VADs (50.9 years). The proportion of female recipients was highest in the ECMO group (32%), followed by the groups on inotropes (27%), IABP (24%), and VAD (19%) (*p* < 0.001). The need for ventilator support also significantly varied between the modalities (*p* < 0.001; the highest was in the ECMO group (24%), the lowest was in the inotrope (2%) and IABP (2%) cohorts, and the VAD group (5%) was in the middle. A significant difference in dialysis dependence was observed (*p* < 0.001): ECMO (18%), inotropes (5%), VAD (7%), and IABP (6%). Prior cardiac surgery was highest in the VAD group (35%), followed by the groups on ECMO (22%), inotropes (12%), and IABP (11%), with a significant difference across groups (*p* < 0.001). Other significant differences in recipient baseline characteristics were observed in history of diabetes, smoking, BMI, PCWP, and mPAP (Table 2).

### 3.5. Survival Outcomes and Postoperative Complications by Bridging Modality

**Figure 3:**
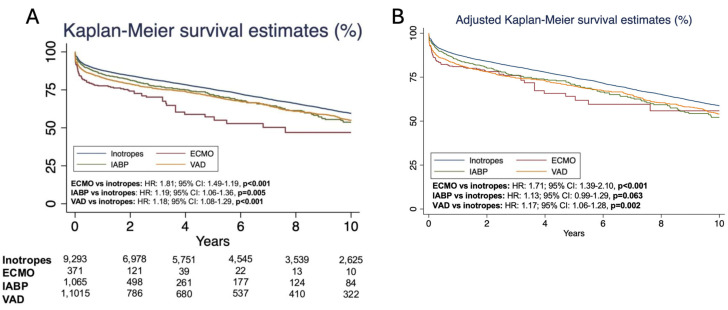
Kaplan–Meier curves representing unadjusted (**A**) and adjusted (**B**) long-term survival in those on inotropes vs. ECMO, IABP, and VAD at the time of heart transplant. CI: confidence interval; HR: hazard ratio; ECMO: extracorporeal membrane oxygenation; IABP: intra-aortic balloon pump; VAD: ventricular assist device.

Survival analysis revealed that inotrope patients had significantly better outcomes across all time points (30 days, 1 year, and 10 years). Thirty-day mortality was lower in those receiving inotropes compared to those on ECMO (HR: 2.98; 95% CI: 2.14–4.16, *p* < 0.001), IABP (HR: 1.51; 95% CI: 1.15–1.99, *p* = 0.004), and VAD (HR: 2.12; 95% CI: 1.66–2.72, *p* < 0.001). One-year mortality followed a similar trend: ECMO (HR: 2.05; 95% CI: 1.63–2.58, *p* < 0.001), IABP (HR: 1.22; 95% CI: 1.03–1.45, *p* = 0.023), and VAD (HR: 1.45; 95% CI: 1.24–1.71, *p* < 0.001). Ten-year overall survival remained significantly higher for inotrope-supported patients, with lower mortality risk compared to ECMO (HR: 1.81; 95% CI: 1.49–2.19, *p* < 0.001), IABP (HR: 1.19; CI: 1.06–1.32, *p* = 0.005), and VAD (HR: 1.18; 95% CI: 1.08–1.29, *p* < 0.001). After adjustment, the HRs for mortality were as follows: ECMO vs. inotropes, 1.71 (95% CI: 1.39–2.10; *p* < 0.001); IABP vs. inotropes, 1.13 (95% CI: 0.99–1.29; *p* = 0.063); and VAD vs. inotropes, 1.17 (95% CI: 1.06–1.29; *p* = 0.02). Unadjusted and adjusted Kaplan–Meier curve for 10-year survival is represented in Figure 3.

Postoperative complications were also lowest in the inotrope group. Risk of stroke in the inotrope group was significantly lower than in the groups on ECMO (OR: 3.44; 95% CI: 2.43–5.19, *p* < 0.001), IABP (OR: 1.55; 95% CI: 1.11–2.16, *p* = 0.009), and VAD (OR: 1.50; 95% CI: 1.07–2.12, *p* = 0.019). Incidence of post-transplant dialysis was also significantly lower in the inotrope group than the ECMO (OR: 3.56; 95% CI: 3.01–4.63, *p* < 0.001) and IABP groups (OR: 1.57; 95% CI: 1.35–1.85, *p* < 0.001). VAD support did not show a statistically significant difference from inotropes in this outcome (OR: 1.10; 95% CI: 0.92–1.31, *p* = 0.292). There were no significant differences in post-transplant pacemaker implantation among the groups. Meanwhile, rejection requiring treatment was significantly less frequent in the inotrope group compared to the ECMO, IABP, and VAD groups (Table 3.).

## 4. Discussion

Our study comprehensively investigated the bridging strategies to heart transplantation (HT), directly comparing outcomes among patients supported with inotropes versus no inotropes and also with those bridged with mechanical circulatory support (MCS) modalities, including extracorporeal membrane oxygenation (ECMO), an intra-aortic balloon pump (IABP), and ventricular assist devices (VADs), using data from the United Network for Organ Sharing (UNOS) national registry encompassing HT patients over the span of two decades. We captured detailed clinical characteristics, perioperative complications, and survival outcomes across both the pre- and post-2018 UNOS heart allocation policy eras, providing a head-to-head comparison of risk profiles.

Our findings reveal that inotropic therapy at the time of transplant is associated with significantly lower 30-day, 1-year, and 10-year mortality and reduced the incidence of complications, such as stroke and post-transplant dialysis when compared with ECMO, IABP, and VAD support. Notably, these benefits were observed without evidence of compromising intermediate-term survival, suggesting the use of inotropes not merely as a fallback option, but rather as a potentially favorable bridging strategy in selected transplant candidates.

While inotropic therapy can aid in bridging patients to transplantation, their continuous use has been associated with complications such as kidney dysfunction, potentially due to the increased systemic vascular resistance impairing renal perfusion, resulting in the need for dialysis post-transplant [9,10,11]. Notably, we observed a significantly lower incidence of post-operative dialysis in the inotrope group, in contrast to earlier concerns that prolonged inotrope use may impair renal perfusion. These favorable short-term outcomes reflect inotropes’ known role in stabilizing hemodynamics in HF [12], reinforcing that, in selected patients, inotropes can serve as an effective bridge to transplantation with a low complication burden.

Consistent with prior studies, patients bridged with ECMO experienced significantly worse short-term outcomes, including nearly three-fold-higher 30-day mortality and markedly increased rates of stroke and renal failure requiring dialysis compared to those supported with inotropes. Although more recent data suggested improved outcomes among ECMO bridged patients following the new 2018 UNOS allocation policy [13], likely due to shorter wait times and more selective use, ECMO remains associated with high perioperative morbidity and early complication risk. Similarly, IABP and VAD support were also associated with higher early mortality and complication rates when compared to inotropic support.

As expected, patients bridged with inotropes in our cohort exhibited clinical profiles consistent with lower-acuity HF. Compared to those supported with ECMO, IABP, or VADs, inotrope recipients had better pre-transplant end-organ function, including lower median creatinine and bilirubin levels, and markedly lower rates of ventilator and dialysis dependence.

Our analysis found that patients bridged to HT with inotropic support demonstrated comparable 1-year post-transplant survival to those supported with VADs, IABP, and ECMO. This outcome challenges the historically negative associations between inotrope use and survival in chronic HF populations and supports their safety in carefully selected transplant candidates. Notably, the early survival advantage of inotropes extended into the long term: at 10 years post-transplant, patients bridged with inotropes had significantly better overall survival compared to all other groups. While our study did not capture waitlist mortality, delisting, or support escalation, it offers a clearer view of post-transplant survival trajectories among patients ultimately transplanted on their initial bridge modality.

In the context of the current UNOS allocation system, where MCS is tied to higher listing priority, these findings may help clarify expectations and guide clinical decision making. Inotrope-bridged patients, while typically listed at lower priority, may still achieve excellent outcomes when timely transplantation is feasible and escalation to MCS is avoided. While these patients may experience longer waitlist times or require closer surveillance for clinical deteriorations, our findings suggest that, when transplantation proceeds successfully, inotropic bridging offers durable outcomes without excess post-transplant risk. These results support the continued role of inotropes as a lower-risk, medically manageable bridging strategy in selected candidates, especially those who are hemodynamically stable with preserved end-organ function.

When it comes to optimizing bridge modality selection in HT candidates, our results inform the nuanced clinical judgement required in choosing a bridge to transplant modality. On one hand, escalating to MCS increases transplant priority in the UNOS system. For example, placing an IABP or other temporary MCS can move a patient to Status 2–3, markedly shortening wait times and reducing the risk of dying or deteriorating on the waitlist [14]. After the 2018 policy change, prioritization of sicker patients has been achieved; nationally, the use of temporary MCS at listing and transplant has surged, contributing to shorter wait times and lower waitlist mortality overall [15,16]. On the other hand, not all advanced HF patients derive a net benefit from early MCS if they can be maintained on inotropes. For a patient who is clinically stable on inotropic support without escalating end-organ dysfunction, it may be prudent to continue that course rather than expose them to risks associated with device-related complications.

Our findings that transplanted inotrope-only patients fare just as well as those on devices support that higher urgency status is not synonymous with better post-transplant outcomes. In clinical practice, this means that an inotrope-dependent patient with adequate perfusion can remain at a lower status, alongside careful monitoring, and still expect excellent transplant results, provided they do not clinically worsen while waiting. Further research is needed on long-term complications of pretransplant inotrope use and the impact of the 2018 UNOS policy change on transplant outcomes.

This study has some potential limitations that require consideration. First, data on the types of inotropes, dose, and duration of therapy were not available and, therefore, could not be used in the study. Additionally, detailed information regarding the specific indications for different MCS devices was not available in the dataset. Furthermore, this is a retrospective analysis of a national registry, and it carries the inherent limitations of the study design. Specifically, determining the precise impact of inotropes is challenging in a retrospective clinical study. Our results are based on the US organ system and may not be generalizable to the rest of the world. Lastly, patient outcomes may have been influenced by other factors that have not been considered in the present analysis.

## 5. Conclusions

Our results show that the continuous use of inotropes after waitlisting is associated with lower 30-day, 1-year, and 10-year mortality compared to ECMO, IABP, and VAD support. Thus, continuation of inotropes to bridge patients to transplantation can be considered a viable option when mechanical support is not feasible.

## Figures and Tables

**Table 1:** Characteristics of the populations at the time of transplantation.

Variable	Inotropes at Tx (*n* = 7168)	No Inotropes at Tx (*n* = 3360)	*p*-Value
Recipient characteristics			
Age, years, mean (SEM)	50.9 ± 14.4	50.8 ± 14.1	0.704
Female gender	2013 (28)	735 (22)	<0.001
Race			
White	4485 (63)	2052 (61)	0.371
Black	1669 (23)	841 (25)	
Other	1014 (14)	467 (14)	
BMI, kg/m^2^	26.2 ± 4.9	27.1 ± 5.2	<0.001
T2D	1915 (27)	868 (25)	0.024
CVD	391 (5)	166 (5)	0.547
Smoking	2435 (34)	1277 (38)	<0.001
Malignancy	605 (8)	220 (7)	0.001
ICD	4703 (66)	2180 (65)	0.464
Dialysis	418 (6)	254 (8)	0.001
IVDU	887 (12)	526 (16)	<0.001
Days on Status 1	0.29 ± 2.1	0.37 ± 2.6	0.059
Days on Status 2	2.4 ± 10.5	2.4 ± 10.1	0.769
Days on Status 3	1.6 ± 12.6	4.5 ± 35.5	<0.001
Days on Status 4	5.7 ± 40.1	18.7 ± 100.7	<0.001
Days on Status 1A	13.1 ± 31.1	36.4 ± 73.6	<0.001
Days on Status 1B	40.1 ± 94.9	114.2 ± 221.9	<0.001
New allocation era	1754 (24)	792 (24)	0.322
LVAD at listing	140 (2)	236 (7)	<0.001
RVAD at listing	47 (1)	41 (1)	<0.001
On ventilator at listing	298 (4)	294 (9)	<0.001
Prior cardiac surgery	1722 (24)	765 (23)	0.005
Creatinine, mg/dL	1.41 ± 0.91	1.39 ± 0.78	0.253
CPRA value	12.3 ± 24.7	11.3 ± 23.5	0.257
Cardiac output, L/min	4.1 ± 1.4	4.1 ± 1.5	0.184
PCWP, mmHg	21.4 ± 8.5	22.9 ± 8.5	<0.001
mPAP, mmHg	30.9 ± 9.8	33.1 ± 9.9	<0.001
IABP at listing	883 (12)	490 (15)	0.001
IABP at Tx	1246 (17)	280 (8)	<0.001
ECMO at listing	198 (3)	132 (4)	0.001
ECMO at Tx	310 (4)	133 (4)	0.383
Donor Characteristics			
Age, years	31.7 ± 11.8	31.2 ± 11.2	0.031
Race			
White	4644 (65)	2168 (65)	0.568
Black	1079 (15)	520 (15)	
Other	1445 (20)	672 (20)	
Female gender	2189 (31)	802 (24)	<0.001
BMI	26.9 ± 5.8	27.1 ± 5.6	0.265
Creatinine, mg/dL	1.4 ± 1.4	1.4 ± 1.4	0.549
T2D	227 (3)	74 (2)	0.002
LVEF, %	61.5 ± 7.2	61.9 ± 7.1	0.004
Ischemic time, hours	3.3 ± 1.1	3.2 ± 1.1	0.466
Distance (miles)	202 ± 255	178 ± 206	<0.001

BMI: body mass index; CPRA: Calculated Panel Reactive Antibody; CVD: cerebrovascular disease; ECMO: extracorporeal membrane oxygenator; IABP: intra-aortic balloon pump; ICD: implantable cardiac defibrillator; IVDU: intravenous drug user; LVAD: left ventricular assist device; LVEF: left ventricular injection fraction; mPAP: mean pulmonary arterial pressure; PCWP: pulmonary capillary wedge pressure; RVAD: right ventricular assist device; SEM: standard error of the mean; T2D: type 2 diabetes; Tx: transplant.

**Table 2:** Baseline characteristics of the populations in the multi-modality bridging analysis.

Variable	Inotropes (*n* = 9330)	ECMO (*n* = 372)	IABP (*n* = 1072)	VAD (*n* = 1027)	*p*-Value
Recipient Characteristics					
Age, years	51.3 ± 14.3	46.3 ± 13.9	54.4 ± 11.9	52.6 ± 11.3	<0.001
Female gender	2554 (27)	118 (32)	255 (24)	197 (19)	<0.001
Race					
White	6210 (67)	221 (59)	707 (66)	720 (70)	<0.001
Black	1891 (20)	96 (26)	233 (22)	236 (23)	
Other	1229 (13)	55 (15)	132 (12)	71 (7)	
BMI, kg/m2	26.6 ± 4.9	27.8 ± 5.8	27.6 ± 4.9	28.1 ± 5.1	<0.001
T2D	1670 (18)	68 (18)	281 (26)	145 (14)	<0.001
CVD	466 (5)	15 (4)	61 (6)	47 (5)	0.414
Smoking	3050 (33)	124 (33)	414 (39)	280 (27)	<0.001
Malignancy	34 (1)	0 (0)	0 (0)	3 (1)	0.179
ICD	6216 (67)	135 (36)	740 (69)	611 (59)	<0.001
Dialysis	456 (5)	67 (18)	68 (6)	76 (7)	<0.001
IVDU	25 (1)	0 (0)	2 (1)	9 (1)	0.644
Days on Status 1	0.1 ± 1.6	5.4 ± 8.7	0.6 ± 4.8	0.1 ± 0.1	<0.001
Days on Status 2	1.3 ± 11.2	2.5 ± 8.1	8.3 ± 14.2	0.2 ± 2.7	<0.001
Days on Status 3	1.9 ± 17.8	1.2 ± 4.9	1.6 ± 8.8	1.1 ± 17.6	<0.001
Days on Status 4	6.6 ± 49.1	10.6 ± 79.5	11.9 ± 65.6	10.9 ± 85.3	<0.001
Days on Status 1A	15.4 ± 39.3	7.4 ± 26.9	10.6 ± 21.6	42.9 ± 64.9	<0.001
Days on Status 1B	56.9 ± 119.6	8.6 ± 53.3	13.3 ± 86.1	158 ± 195	<0.001
New allocation era	1348 (14)	284 (76)	647 (60)	36 (4)	
On ventilator at listing	178 (2)	89 (24)	22 (2)	52 (5)	<0.001
Prior cardiac surgery	1087 (12)	81 (22)	120 (11)	363 (35)	<0.001
Creatinine, mg/dL	1.4 ± 0.9	1.5 ± 1.2	1.5 ± 1.2	1.4 ± 0.7	<0.001
CPRA value	12.8 ± 24.9	13.9 ± 26.7	10.7 ± 23.1	17.5 ± 30.7	<0.001
Cardiac output, L/min	4.4 ± 1.5	4.5 ± 2.1	4.3 ± 1.4	4.8 ± 1.5	<0.001
PCWP, mmHg	19.7 ± 8.5	20.7 ± 10.1	20.9 ± 8.8	18 ± 5 9.6	<0.001
mPAP, mmHg	42.9 ± 14.1	45.1 ± 22.6	44.3 ± 14.5	41.3 ± 14.3	<0.001
Donor Characteristics					
Age, years	31.5 ± 11.8	29.5 ± 10.2	32.1 ± 10.7	30.9 ± 11.5	<0.001
Race					
White	6129 (66)	217 (58)	669 (62)	723 (70)	<0.001
Black	1329 (14)	81 (22)	198 (18)	149 (15)	
Other	1872 (20)	74 (20)	205 (20)	155 (15)	
Female gender	2868 (31)	87 (23)	269 (25)	231 (22)	<0.001
BMI	26.8 ± 5.7	27.4 ± 5.8	27.5 ± 6.1	27.1 ± 5.5	0.006
Creatinine, mg/dL	1.4 ± 1.3	1.4 ± 1.5	1.6 ± 1.7	1.3 ± 1.1	<0.001
T2D	275 (3)	9 (2)	37 (3)	25 (2)	0.676
LVEF, %	61.6 ± 7.3	61.7 ± 6.8	61.6 ± 6.9	61.9 ± 7.6	0.002
Ischemic time, h	3.2 ± 1.1	3.5 ± 0.9	3.4 ± 0.9	3.2 ± 1.7	<0.001
Distance (mi)	183 ± 216	252 ± 193	249 ± 208	146 ± 197	<0.001

SEM: standard error of the mean; BMI: body mass index; T2D: type 2 diabetes; CVD: cerebrovascular disease; ICD: implantable cardiac defibrillator; IVDU: intravenous drug user; CPRA: Calculated Panel Reactive Antibody; PCWP: pulmonary capillary wedge pressure; mPAP: mean pulmonary arterial pressure; LVEF: left ventricular injection fraction.

**Table 3:** Outcomes of bridging strategies to heart transplantation: inotropes vs. ECMO, IABP, and VAD.

Outcome	Comparison	HR	95% CI	*p*-Value
Overall mortality	ECMO vs. inotropes	1.81	1.49–2.19	<0.001
	IABP vs. inotropes	1.19	1.06–1.36	0.005
	VAD vs. inotropes	1.18	1.08–1.29	<0.001 <0.001
1-Year mortality	ECMO vs. inotropes	2.05	1.63–2.58	
	IABP vs. inotropes	1.22	1.03–1.45	0.023
	VAD vs. inotropes	1.45	1.24–1.71	<0.001
30-Day mortality	ECMO vs. inotropes	2.98	2.14–4.16	<0.001
	IABP vs. inotropes	1.51	1.15–1.99	0.004
	VAD vs. inotropes	2.12	1.66–2.72	<0.001
Complication	Comparison	OR	95% CI	*p*-value
Stroke	ECMO vs. inotropes	3.55	2.43–5.19	<0.001
	IABP vs. inotropes	1.55	1.11–2.16	0.009
	VAD vs. inotropes	1.50	1.07–2.12	0.019
Dialysis	ECMO vs. inotropes	3.56	3.01–4.63	<0.001
	IABP vs. inotropes	1.57	1.35–1.85	<0.001
	VAD vs. inotropes	1.10	0.92–1.31	0.292
Pacemaker	ECMO vs. inotropes	0.51	0.21–1.24	0.135
	IABP vs. inotropes	0.98	0.65–1.46	0.906
	VAD vs. inotropes	0.71	0.45–1.14	0.192
Rejection requiring treatment	ECMO vs. inotropes	1.66	1.35–2.04	<0.001
	IABP vs. inotropes	1.29	1.13–1.46	<0.001
	VAD vs. inotropes	1.20	1.05–1.36	0.006

CI: confidence interval; HR: hazard ratio; OR: odds ratio.

## Data Availability

The data that support the findings of this study are available on request from the corresponding author. The data are not publicly available due to privacy or ethical restrictions.

## Abbreviations

The following abbreviations are used in this manuscript:

BMI	body mass index
CPRA	Calculated Panel Reactive Antibody
CVD	cerebrovascular disease
ECMO	extracorporeal membrane oxygenation
HF	heart failure
HT	heart transplantation
IABP	intra-aortic balloon pump
IV	intra-venous
ICD	implantable cardioverter-defibrillator
LVAD	left ventricular assist device
MCS	mechanical circulatory support
UNOS	United Network for Organ Sharing
RVAD	right ventricular assist device
